# Research on Driver Fatigue Detection in Real Driving Environments Based on Semi-Dry Electrodes with Automatic Conductive Fluid Replenishment

**DOI:** 10.3390/s25216687

**Published:** 2025-11-01

**Authors:** Fuwang Wang, Yuanhao Zhang, Weijie Song, Xiaolei Zhang

**Affiliations:** School of Mechanic Engineering, Northeast Electric Power University, Jilin 132012, China; 20152622@neepu.edu.cn (F.W.); 20142561@neepu.edu.cn (Y.Z.); 13844244845@sohu.com (W.S.)

**Keywords:** driving fatigue, semi-dry electrode, EEG, transfer learning

## Abstract

Driving fatigue poses a serious threat to road safety. To detect fatigue accurately and thereby improve vehicle safety, this paper proposes a novel semi-dry electrode with the ability to automatically replenish the conductive fluid for monitoring driving fatigue. This semi-dry electrode not only integrates the advantages of both wet and dry electrodes but also incorporates an automatic conductive fluid replenishment mechanism. This design significantly extends the operational lifespan of the electrode while mitigating the limitations of manual replenishment, particularly the risk of signal interference. Additionally, this study adopts a transfer learning approach to detect driving fatigue by analyzing electroencephalography (EEG) signals. The experimental results indicate that this method effectively addresses the issue of data sparsity in real-time fatigue monitoring, overcomes the limitations of traditional algorithms, shows strong generalization performance and cross-domain adaptability, and achieves faster response times with enhanced accuracy. The semi-dry electrode and transfer learning algorithm proposed in this study can provide rapid and accurate detection of driving fatigue, thereby enabling timely alerts or interventions. This approach effectively mitigates the risk of traffic accidents and enhances both vehicle and road traffic safety.

## 1. Introduction

With the rapid progress of modern industrial technology, driving has become the primary mode of transportation for the general population. However, driving fatigue reduces reaction speed and the capacity to evaluate road conditions, thereby constituting a major contributing factor to traffic accidents. Consequently, the real-time and accurate detection of driving fatigue is crucial for ensuring road safety.

To achieve real-time and precise detection of driving fatigue, numerous methods have been proposed both domestically and internationally. Traditional approaches include facial feature analysis, vehicle driving parameter assessment, and eye movement-based evaluation. Nonetheless, these methods face limitations under real-world driving conditions. For instance, Li et al. [1] assessed driving fatigue by inputting features such as eyelid aspect ratios, lip and eyebrow metrics, and head posture angles into a decision network. However, under conditions such as nighttime driving or variable lighting, accurate collection of facial features is challenging, significantly reducing detection accuracy. Li et al. [2] employed steering wheel angle data for fatigue assessment. Given the variability in individual driving habits, alterations in certain vehicle operating parameters may not reliably reflect driving fatigue, thereby constraining the generalizability of this approach and increasing the likelihood of false positives. Xu et al. [3] analyzed eye movement trajectories, fixation duration, and pupil area to assess fatigue. Yet, external factors such as vehicle vibrations and direct light exposure may lead to erroneous assessments, and extended eye-tracking measurements may cause ocular discomfort. With advances in EEG signal analysis and acquisition devices, researchers worldwide have increasingly utilized EEG signals to evaluate brain fatigue. As a physiological signal that directly reflects brain activity, EEG offers inherent advantages in sensitivity and real-time performance compared to traditional methods. Recently, Wang et al. [4] detected driving fatigue by extracting the 14~32 Hz subband from EEG signals and applying multifractal detrended fluctuation analysis (MF-DFA). These advantages make EEG a more reliable and objective indicator for fatigue detection, especially in dynamic and noisy driving environments where behavioral and ocular signals are easily confounded. Accordingly, this study employs driver EEG signal detection as a method for assessing fatigue.

The main sensor types for EEG signal acquisition are wet, dry, and semi-dry electrodes. For example, Qin et al. [5] employed Neuroscan’s SynAmps 64-channel amplifier (Neuroscan, Compumedics USA, Charlotte, NC, USA) to collect EEG signals. This wet electrode relies on conductive gel to lower skin impedance and improve signal acquisition quality, resulting in high-quality, stable EEG recordings. However, prolonged use of conductive gel may cause skin discomfort, allergic reactions, and gradual gel drying, which significantly degrades signal extraction performance. Consequently, experimenters must replenish the conductive gel promptly to maintain signal quality. Wang et al. [6] developed a novel elastic dry electrode to address skin discomfort and the complexity of wet electrode placement. This design provides ease of use, a simple structure, and ease of fabrication. Nevertheless, without conductive gel as an interface, skin impedance is not effectively reduced, leading to reduced signal quality and lower resistance to interference compared with wet electrodes. To overcome the limitations of both dry and wet electrodes, Wang et al. [7] proposed a portable semi-dry electrode integrating the advantages of both types. Liu et al. [8] proposed a hydrogel-based semi-dry electrode capable of stable EEG acquisition for up to 10 h, effectively addressing the volatility of conductive gels and the limited operating duration of wet electrodes. However, its dependence on ambient humidity to maintain conductivity restricts long-term use under complex environmental conditions. Zhu et al. [9] developed a flexible semi-dry electrode composed of reduced graphene oxide and polyurethane sponge, providing low impedance, high conformability, and improved signal stability and comfort. Nevertheless, this design still requires manual replenishment of the conductive medium, and its automation and long-term stability remain to be optimized. Previously developed semi-dry electrodes, such as those proposed by Yao [10], Wang [11], and Li [12], support manual replenishment of the conductive fluid but lack the capability for automatic replenishment. Consequently, manual intervention is still required, during which the physical manipulation involved in pressing or pulling the electrode may introduce noise interference, thereby deteriorating the quality of EEG signal acquisition. While traditional wet electrodes struggle to maintain consistent electrode positioning during gel replenishment. Existing semi-dry electrodes remain constrained by limited operational duration and the need for frequent manual replenishment, making them unsuitable for long-term, practical applications such as continuous driving fatigue monitoring. To overcome the limitations of conventional semi-dry electrodes, namely limited operational duration and high dependence on manual conductive fluid replenishment, this study develops a semi-dry electrode with automatic conductive fluid replenishment. This design effectively extends the operational lifespan of the electrode, eliminates labor-intensive manual replenishment, and minimizes dependence on human intervention. Accordingly, this study utilizes the semi-dry electrode with automatic conductive fluid replenishment to acquire EEG signals for evaluating driving fatigue.

Currently, traditional algorithms, including backpropagation neural networks (BPNNs), random forests (RFs), convolutional neural networks (CNNs), and support vector machines (SVMs), are commonly employed to assess driving fatigue. However, these approaches exhibit inherent limitations. Chen et al. [13] employed a BPNN combined with a time-accumulation effect model to mitigate detection errors caused by facial expressions in drowsy driving. However, the training of BPNNs typically necessitates large volumes of labeled data, and in tasks such as drowsy driving detection, limited sample sizes may result in overfitting. Zhou et al. [14] proposed an improved random forest-based fatigue detection method incorporating multi-physical characteristics, which demonstrated notable improvements in detection reliability and accuracy. Nevertheless, this approach retains the limitations of traditional random forest methods: it exhibits strong dependence on conventional machine learning techniques, has limited cross-domain generalizability, and requires the construction of multiple decision tree models. Small sample sizes further reduce prediction accuracy, thereby constraining the model’s ability to generalize. Wang et al. [15] applied the Gauss-Annex-Difference Field-Convolutional Neural Network (GADF-CNN) to extract fatigue features from EEG signals. Although GADF can capture subtle fatigue features in EEG signals that may be obscured by environmental influences, CNNs often exhibit scene dependency, leading to failure under changes in environment or subjects. Additionally, CNN training demands substantial labeled data, limiting applicability in sample-scarce domains. You et al. [16] utilized an SVM to establish a fatigue detection model. However, this method requires manual feature extraction and labeling, restricting the use of unlabeled data and increasing dependence on human intervention. Its performance deteriorates under environmental changes or domain shifts, indicating limited cross-domain generalizability.

To address the limitations inherent in traditional algorithms, this study employs transfer learning to assess driving fatigue. This approach exploits similarities between domains or tasks, allowing knowledge acquired in one domain to be transferred to another, and thereby enhancing the model’s generalization and cross-domain adaptability. Even under conditions of limited data in driving fatigue detection, knowledge from a source-domain model can be transferred to the target domain, mitigating data scarcity challenges. Furthermore, the pre-trained model can be fine-tuned to specific requirements, enabling rapid development of a highly generalizable model. Vafaei et al. [17] conducted a systematic review on the use of Transformer architectures, data augmentation, and transfer learning in EEG analysis, emphasizing that transfer learning can effectively mitigate generalization issues under limited data conditions. Their findings demonstrate strong feasibility in cross-subject and cross-task EEG feature transfer, offering valuable guidance for EEG-based feature recognition.

## 2. Materials and Methods

### 2.1. The Production of Semi-Dry Electrode with Automatic Electrolyte Replenishment

#### 2.1.1. Structural Design

**Figure 1:**
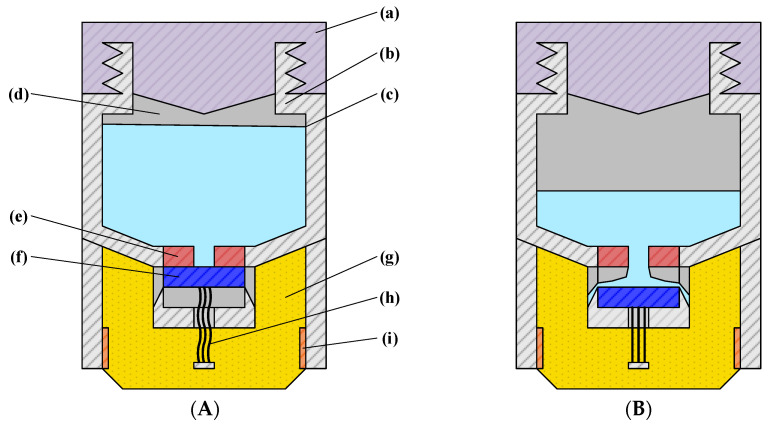
The structure of the semi-dry electrode. (**A**) Electrode in non-operating state: (a) Electrode cover; (b) Electrode main body housing; (c) Conductive liquid; (d) Conductive liquid chamber; (e) Magnet 1; (f) Magnet 2; (g) PVA sponge; (h) Degreased hair; (i) Copper sheet. (**B**) Electrode in operating state.

**Figure 2:**
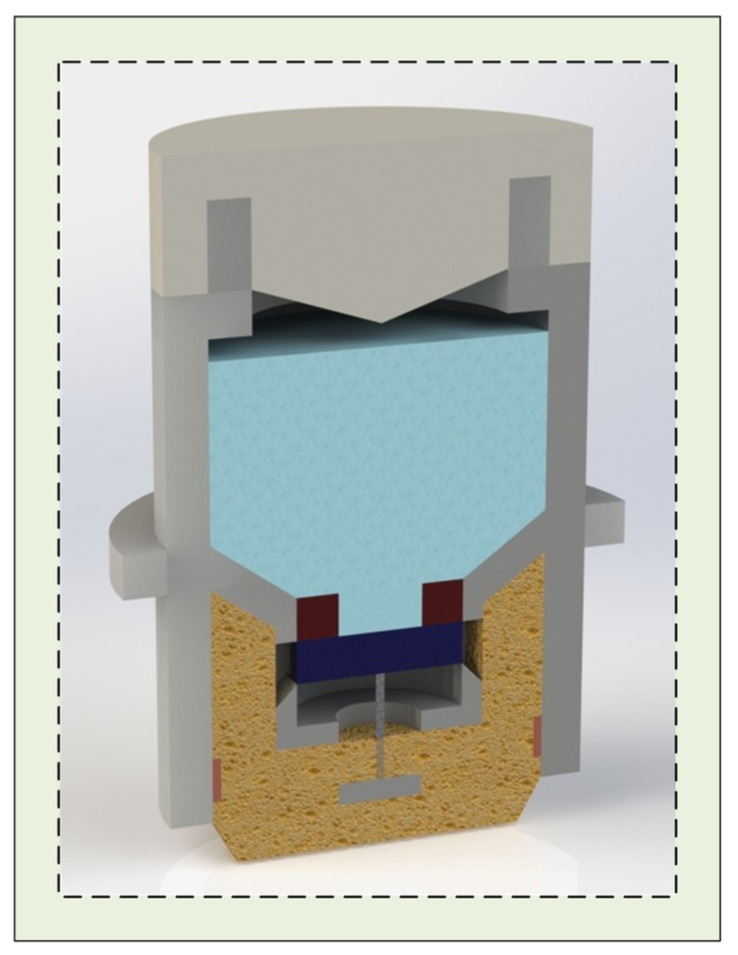
A half-sectional view of the assembled electrode.

**Figure 3:**
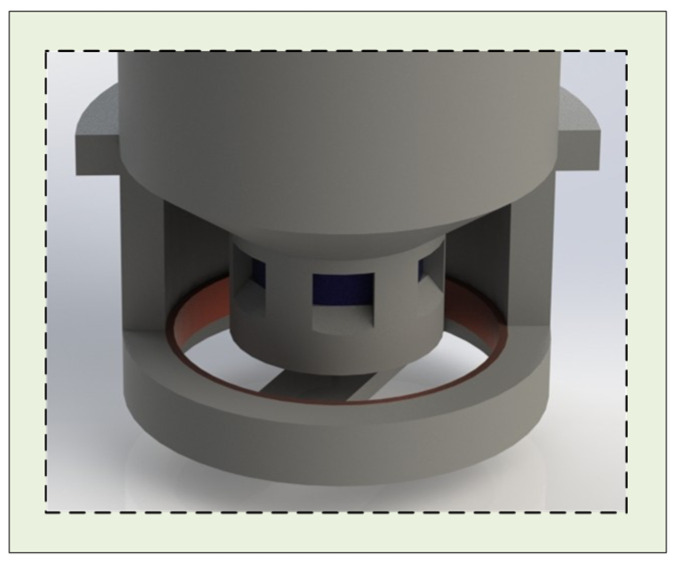
The key structural arrangement of the electrode magnets.

In this design, magnet 1 is fixed to the electrode body housing, while magnet 2 is constrained by the support structure to move only along the vertical axis. The attraction and separation between the two magnets regulate the flow of conductive liquid into the underlying PVA sponge. One end of the degreased hair is anchored to the center of magnet 2, with the other end attached to a longitudinal crossbeam connected to the electrode housing (Figure 1, Figure 2 and Figure 3). As the PVA sponge dries, the degreased hair contracts, pulling magnet 2 downward and separating it from magnet 1, thereby allowing conductive liquid to flow through the central hole of magnet 1 into the sponge. As the sponge becomes saturated, the hair absorbs moisture, expands, and loses tension, enabling the magnets to re-adhere and stop further liquid flow. This mechanism completes the automatic replenishment cycle of the semi-dry electrode. When the conductive liquid in the chamber is depleted, the electrode cap is first rotated counterclockwise to open it, refilled to the indicated level (Figure 1A), and then rotated clockwise to lock it. The plug-like structure of the cap compresses residual air in the chamber upon locking, pressurizing the liquid and ensuring consistent flow to the lower structures under varying environmental conditions.

This structure restricts the vertical movement of the magnet connected to the degreased hair, preventing deflection or horizontal displacement. During conductive liquid replenishment, the two magnets (Figure 1A (e,f)) separate, allowing liquid to flow from the chamber into the underlying PVA sponge. The porous configuration ensures smooth fluid transfer, while the ramped design adjacent to the openings accelerates the flow compared to a flat surface.

#### 2.1.2. Manufacturing of Electrodes

**Figure 4:**
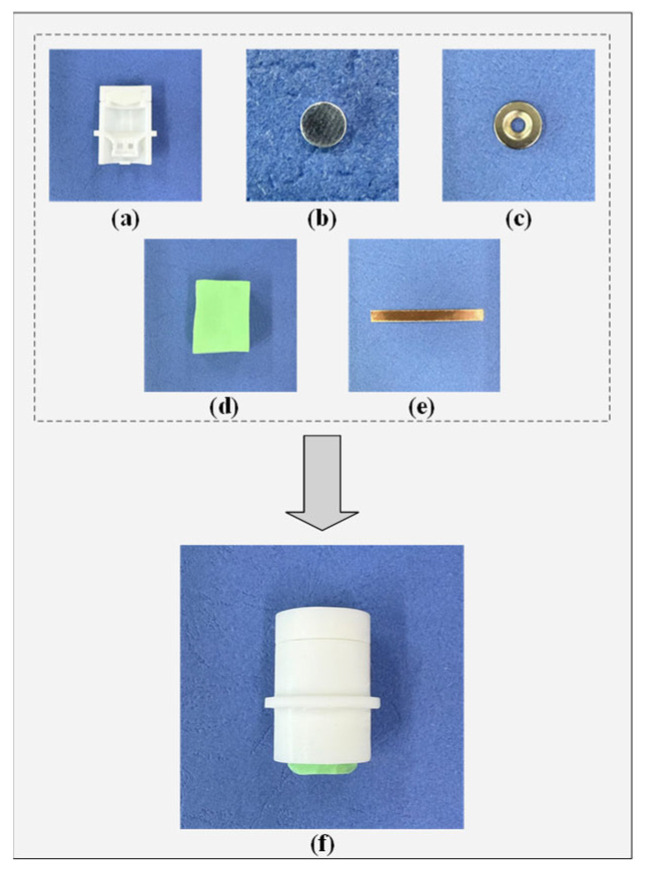
The fabrication process of the semi-dry electrode developed in this study, featuring automatic conductive electrolyte replenishment. (**a**) Half-section view of the electrode housing; (**b**) Magnet; (**c**) Magnet with a central circular hole; (**d**) PVA sponge; (**e**) Copper sheet; (**f**) Prefabricated electrode.

The fabrication process of the semi-dry electrode proceeds as follows Figure 4. First, the main body of the electrode is modeled using software and subsequently 3D-printed to produce the electrode cap and housing. Two magnets are installed at designated positions on the housing. One end of the degreased hair is secured to the center of the solid magnet, while the other end is attached to the center of the lower crossbeam within the housing. A copper sheet is flattened, cut into a long rectangular strip, and bent into a ring matching the inner circumference of the electrode shell, then adhered to the inner wall. Finally, a PVA sponge is placed in the designated compartment, completing the assembly of the semi-dry electrode.

The total weight of the proposed electrode, including the conductive fluid, is approximately 12 g. During operation, the electrode is fixed to the head using an elastic band that applies a pressure of approximately 1–1.5 N, ensuring that the sponge tip maintains stable contact with the scalp.

#### 2.1.3. Impedance Test of Electrode

**Figure 5:**
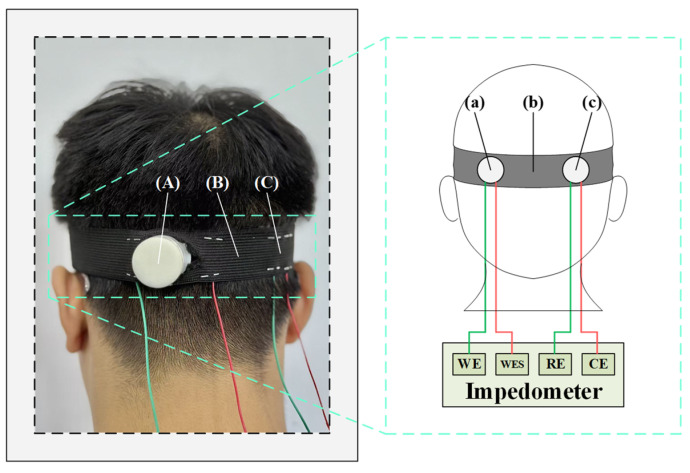
The schematic diagram of the electrode impedance test. (A) Semi-dry electrode; (B) Elastic bandage; (C) Ag/AgCl wet electrode; (a) Semi-dry electrode; (b) Elastic bandage; (c) Ag/AgCl wet electrode.

During EEG signal acquisition, the contact impedance between the electrodes and the skin directly affects signal quality. Lower contact impedance reduces noise and enhances the fidelity of the recorded signals. The electrode impedance testing experiment requires additional materials, including syringes, bandages, Ag/AgCl electrodes, conductive gel, and abrasive paste. Before the commencement of testing, an appropriate amount of abrasive paste was applied to the O1 channel of the subject’s scalp, after which a uniform layer of conductive gel was applied. The semi-dry and wet electrodes are positioned approximately 2 cm apart and secured to the subject’s head using elastic bandages. The circuit diagram for this electrode impedance test is presented in Figure 5.

#### 2.1.4. Electrode Life Test

**Figure 6:**
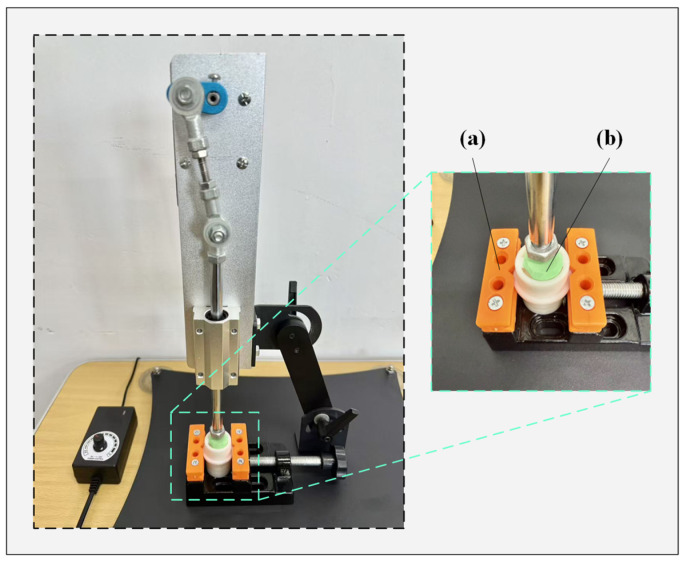
The experimental setup for electrode lifetime testing. (a) Fixture; (b) Electrode.

This experiment assesses the mechanical stability of the electrode under repeated compression. To evaluate its operational lifespan, a compression device applies 100 cycles per minute. The test consists of seven stages, lasting 90 min in total. At the end of each stage, the electrode’s height and elasticity are precisely measured. The electrode life testing setup is shown in Figure 6.

### 2.2. Experiment

#### 2.2.1. Subjects

This study recruited 10 volunteers with a mean age of 30 ± 4.5 years. All participants had previously completed standardized training in driving safety. To ensure the reliability of the data, participants were instructed to maintain sufficient sleep on the day of the experiment and to refrain from staying up late, consuming alcohol, taking medication, or engaging in vigorous physical activity. Before the experiment formally commenced, each participant was randomly assigned a unique identifier ranging from “1” to “10.” Inclusion required that all participants fully understood the study objectives and procedures and voluntarily provided written informed consent. The research protocol was conducted in strict accordance with the ethical principles of the Declaration of Helsinki and was approved in advance by the Ethics Committee of the Affiliated Hospital of Northeast Electric Power University.

#### 2.2.2. Experimental Paradigm

The data acquisition system employed in this experiment utilizes a single EEG channel. According to Wang et al. [18], signals recorded from the occipital region can effectively reflect variations in mental fatigue. Consequently, the O1 lead in the occipital area was selected as the focus of this study. Furthermore, previous investigations by Shahbakhti et al. [19] and Houshmand et al. [20] have demonstrated that EEG information obtained from a single lead is sufficient to support experimental procedures and maintain high accuracy in the results.

**Figure 7:**
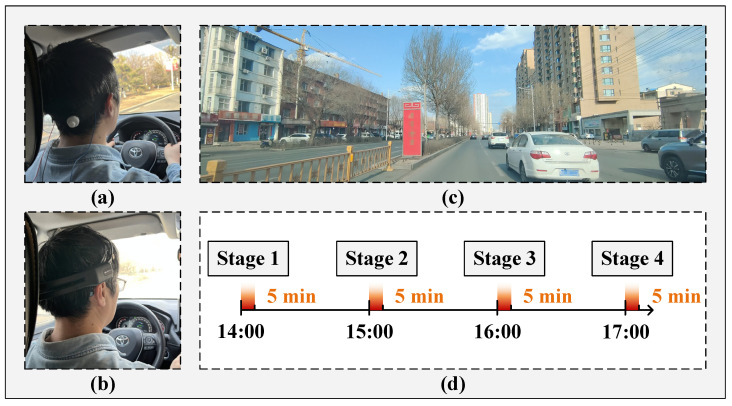
The experimental workflow diagram. (**a**) The electrode device proposed in this paper; (**b**) Emotiv electrode device; (**c**) Schematic diagram of road traffic conditions; (**d**) Schematic diagram of EEG data acquisition process.

The study was carried out in a real driving environment during the period from 14:00 to 17:00. EEG data collection was organized into four stages: Stage 1 (14:00–14:05), Stage 2 (15:00–15:05), Stage 3 (16:00–16:05), and Stage 4 (17:00–17:05). In each phase, EEG signals were recorded from the O1 channel for a duration of 5 min. A 60 min interval was maintained between consecutive sessions to ensure adequate recovery and temporal separation of the recordings. During each EEG recording session, participants were instructed to subjectively assess their fatigue level using the Karolinska Sleepiness Scale (KSS) [21], and the corresponding scores were simultaneously recorded for further analysis. Each participant underwent two experimental conditions: one employing Emotiv electrodes for EEG signal acquisition and the other utilizing semi-dry electrodes. Both electrode types were positioned in accordance with the international 10–20 system, and signals were recorded at a sampling frequency of 128 Hz. Prior to data acquisition, researchers provided participants with a comprehensive briefing covering the study objectives, procedures, and equipment operation. The overall experimental workflow is illustrated in Figure 7.

### 2.3. Methods

Transfer learning is a machine learning approach that exploits similarities among data, tasks, or models to apply knowledge acquired in a source domain to a target domain. It allows models trained on related labeled data to generalize to unlabeled datasets. Domain adaptation and domain generalization techniques within transfer learning mitigate performance degradation arising from differences in data distributions. Fine-tuning pre-trained models with domain-specific data enables the development of more universally applicable models. Moreover, adaptive mechanisms in transfer learning facilitate personalized modeling, addressing the diverse requirements of individual users.

In general, the goal of transfer learning is to effectively perform tasks in a target domain by utilizing knowledge and experience acquired from related source domains.

Transfer learning can be formally defined as follows: Given a source domain $D_{s}$ and a target domain $D_{t}$, where $D=\left\{X,P(X)\right\}$, and a source task $T_{s}$ and a target task $T_{t}$, where $T=\left\{Y,(P(Y|X)\right\}$, the objective of transfer learning—when $D_{s}\neq D_{t}$—is to leverage information from both $D_{s}$ and $D_{t}$ to learn the conditional probability distribution $P(Y_{t}|X_{t})$ in the target domain $D_{t}$.

The training process of a transfer learning model generally consists of three stages. First, a high-performance source domain model is trained using a large amount of labeled source domain data. Second, the general knowledge acquired by the source domain model is transferred to the target domain model through transfer learning techniques. Third, the target domain model is fine-tuned with a small set of labeled target domain data to further optimize its performance.

#### Deep Domain Confusion (DDC)

**Figure 8:**
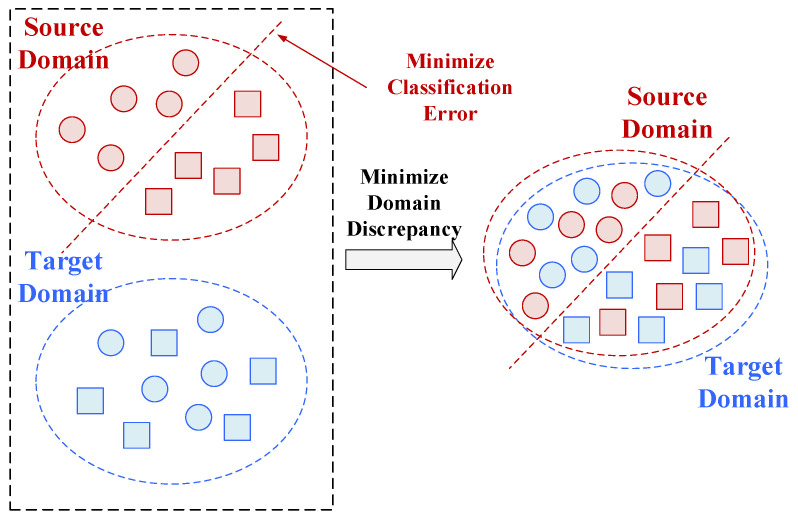
A schematic diagram of DDC.

DDC is a feature-driven transfer learning approach that aims to minimize the discrepancy between feature distributions in the source and target domains, thereby facilitating effective learning for the target task. Specifically, the network is initially trained on the source domain while simultaneously aligning the feature distributions of both domains. Following alignment, the target domain model acquires feature representations akin to those of the source domain. These features are then fine-tuned to obtain an optimized model suitable for the target domain (Figure 8). Furthermore, a limited number of labeled target domain samples can be incorporated alongside source domain data to enhance generalization and predictive accuracy.

Maximum Mean Discrepancy (MMD) is a widely used metric in transfer learning for assessing the similarity between source and target domains. It quantifies the discrepancy by computing the MMD distance between features from both domains, which is then incorporated as a domain adaptation loss. By minimizing this distance, the distributional difference between the source and target domains can be effectively reduced.

**Figure 9:**
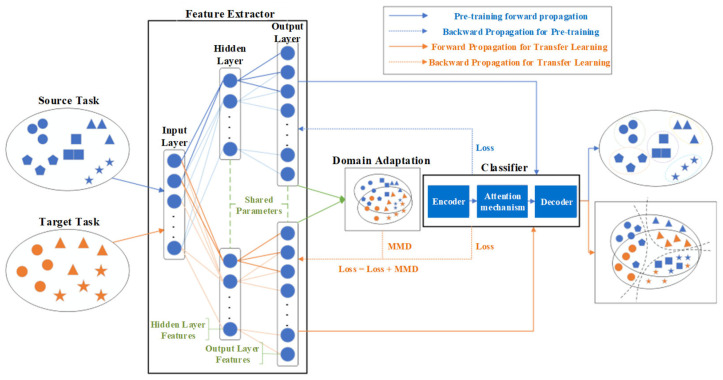
Implementation process of transfer learning algorithm. Blue symbols represent data samples from the source domain, while orange symbols represent samples from the target domain. Different shapes (circles, squares, triangles, and stars) corre-spond to different feature categories or classes.

Figure 9 presents a flowchart illustrating the implementation process of the transfer learning algorithm.

In the process of transfer learning, incorporating the MMD into the model’s loss function helps guide the model to simultaneously learn the feature distributions of both the source domain and the target domain, thereby achieving cross-domain adaptation and feature alignment through optimized training.

For instance, the Multi-domain Aggregation Transfer Learning with Domain-Class Prototype (MATL-DC) framework proposed by Li et al. [22] employs the MMD to aggregate features from multiple domains, thereby enhancing the representational capacity of emotional EEG signals and achieving performance superior to that of conventional methods. Additionally, the Transfer Discriminative Dictionary Pair Learning (TDDPL) method introduced by Ruan et al. [23] incorporates MMD into dictionary learning, enabling effective cross-subject EEG emotion classification and demonstrating MMD’s efficacy in processing EEG signals. Collectively, these studies highlight the significant advantages of MMD in EEG-based transfer learning, as it effectively aligns feature distributions across different domains, thereby improving model generalization and overall performance. Therefore, integrating the MMD into the model’s loss function during the transfer learning process facilitates simultaneous learning of feature distributions from both the source and target domains, thereby enabling effective cross-domain adaptation and feature alignment through optimized training.

The core principle of MMD is to quantify the difference between two distributions by comparing the variance of their expected values over a class of functions in the Reproducing Kernel Hilbert Space (RKHS). MMD is defined based on RKHS functions, representing the discrepancy between two distributions as the maximum mean function within this space. Specifically, for two distributions s and t, MMD is defined as:

(1) $$MMD^{2}\left(s,t\right)=\underset{{\left\|\phi \right\|}_{H}\leq 1}{sup}{\left\|E_{x^{s}\sim s}[\phi (x^{s})]-E_{x^{t}\sim t}[\phi (x^{t})]\right\|}_{H}^{2},$$

Here, ϕ(·) denotes a mapping function that projects the original data into the RKHS. The constraint ${\left\|\phi \right\|}_{H}\leq 1$ defines a set of functions confined within the unit ball of the reproducing kernel Hilbert space. $E_{x^{s}\sim s}\left[\phi (\cdot )\right]$ represents the expected value with respect to the distribution. In practice, since the true distributions of the source and target domains are unknown, empirical estimates are typically employed. Given the source domain sample set $D_{s}=\{x_{i}^{s}{\}}_{i=1}^{M}$ and the target domain sample set $D_{t}=\{x_{i}^{t}{\}}_{i=1}^{N}$, the empirical MMD is expressed as:

(2) $${\hat{MMD}}^{2}\left(D_{s},D_{t}\right)={\left\|\frac{1}{M}\sum_{i=1}^{M}\phi \left(x_{i}^{s}\right)-\frac{1}{N}\sum_{j=1}^{N}\phi \left(x_{j}^{t}\right)\right\|}_{H}^{2},$$

Here M and N represent the numbers of samples in the source and target domains, respectively. The mapping function ϕ(·) corresponds to the feature transformation associated with the kernel function, expressed as $k(x^{s},x^{t})=\left\langle \phi (x^{s}),\phi (x^{t})\right\rangle$. Typically, the kernel function $k(x^{s},x^{t})$ is constructed as a convex combination of L basis kernels $k_{l}(x^{s},x^{t})$.

(3) $$k(x^{s},x^{t})=\sum_{l=1}^{L}\beta_{l}k_{l}(x^{s},x^{t}),s.t.\beta_{l}\geq 0,\sum_{l=1}^{L}\beta_{l}=1,$$

In this study, a Gaussian kernel function is adopted as the base kernel, defined as follows:

(4) $$k(x,y)=\exp(-\frac{{\left\|x-y\right\|}^{2}}{2\sigma^{2}}),$$

Here, σ denotes the width parameter of the Gaussian kernel.

During transfer learning, incorporating MMD into the model’s loss function facilitates adaptive training between the source and target domains.

The front section of the model functions as a feature extractor, with its parameters kept frozen during the transfer learning process. As summarized in Table 1, the feature extraction module primarily comprises one-dimensional convolutional and max-pooling layers. To capture EEG signal characteristics across multiple temporal scales, the model integrates both small-window and large-window convolutional kernel designs. The ReLU function is utilized as the activation function for all layers, and a Dropout layer with a probability of 0.5 is applied after each pooling layer to alleviate overfitting.

During the transfer learning phase, only the parameters of the classifier’s seq2seq module are updated. The classifier consists of an encoder, an attention layer, and a decoder (Table 2). To adapt the model to the three-class classification task of fatigued driving, the output layer is modified from Dense(5) to Dense(3). Furthermore, the MMD loss is incorporated into the overall loss function to promote feature alignment between EEG representations from the sleep domain and those from the fatigued driving domain.

## 3. Results

### 3.1. Electrode Properties

The performance of electrodes is largely governed by two key parameters: service life and skin–electrode contact impedance. To comprehensively evaluate these parameters, dedicated experiments were conducted to measure contact impedance and assess electrode longevity. The corresponding results are presented below.

#### 3.1.1. Contact Impedance of Electrodes

**Figure 10:**
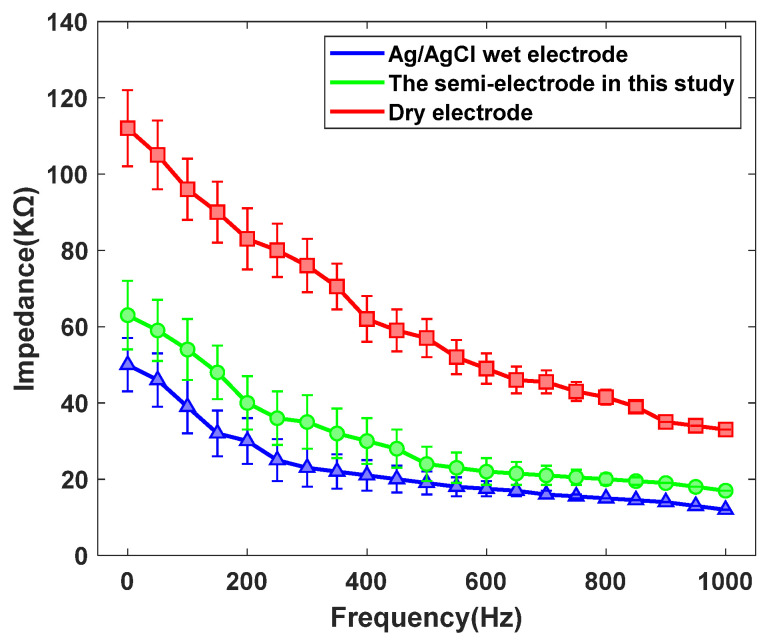
The contact impedance curves of the three types of electrodes with human skin.

As illustrated in Figure 10, the dry electrode demonstrates the highest contact impedance, exhibiting a substantial difference from the other two electrode types. By contrast, the wet electrode achieves the lowest impedance, while the semi-dry electrode shows intermediate values, slightly higher than those of the wet electrode. The elevated impedance of the dry electrode is primarily attributed to the absence of an external conductive medium at the skin–electrode interface, where only limited perspiration and moisture provide conductivity. In comparison, both wet and semi-dry electrodes employ conductive gels that rapidly establish an electrolyte channel, thereby reducing impedance. Although the impedance of semi-dry electrodes remains marginally above that of wet electrodes, they offer distinct advantages, including automatic conductive fluid replenishment, extended operational lifespan, and enhanced wearing comfort, making them more suitable for diverse application scenarios.

#### 3.1.2. Service Life of Electrodes

The quality of EEG signals collected during experimental procedures is closely influenced by the contact pressure between the electrodes and the skin. With prolonged use, the internal support structures of the electrodes are subjected to repeated mechanical stress, gradually leading to fatigue and structural deterioration. This degradation in turn diminishes electrode performance, ultimately compromising the reliability and accuracy of EEG signal acquisition. Consequently, lifespan testing of electrodes constitutes a critical and indispensable component of performance evaluation.

**Figure 11:**
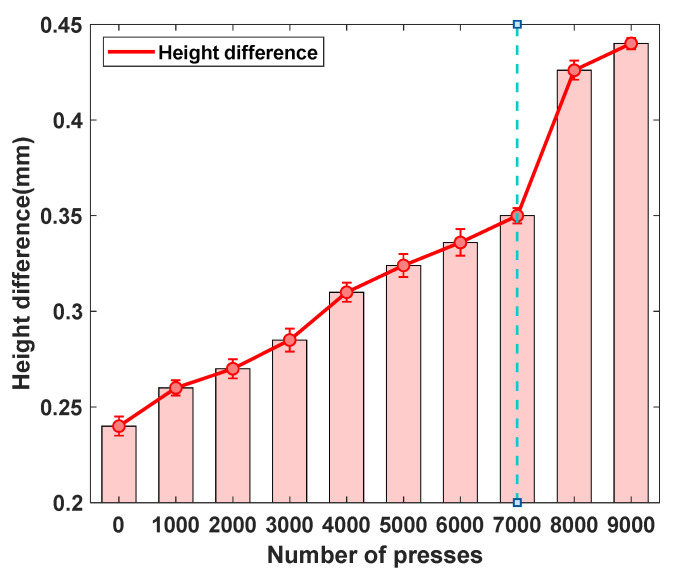
The lifespan curve of the semi-dry electrodes developed in this study.

As illustrated in Figure 11, when the number of force applications remains below 7000, the increase in electrode height difference progresses at a relatively gradual rate. In contrast, once the number of force applications exceeds 7000, the rate of increase in height difference accelerates markedly, demonstrating a pronounced trend of structural deterioration. These results indicate that the performance of the electrodes experiences a critical decline beyond approximately 7000 loading cycles.

### 3.2. Performance Analysis of Migration Models

The inherently low amplitude of EEG signals makes them highly susceptible to external disturbances such as eye blinks, muscle contractions, and subtle body movements, which may introduce considerable experimental errors. Consequently, preprocessing is an essential step to enhance signal reliability and analytical accuracy. In this study, a 0.5–45 Hz bandpass filter was first applied to the raw EEG data to suppress baseline drift and power-line interference. Subsequently, Independent Component Analysis was employed to identify and remove artifacts arising from electrooculographic activity, electromyographic interference, and environmental noise [24], thereby yielding cleaner and more stable EEG recordings.

#### 3.2.1. Model Parameter Selection

This study employs the Sleep-EDF dataset [25], consistent with that used in [26], to train the Sleep EEG Net model as the pre-trained network. The Sleep-EDF dataset consists of two subsets: Sleep-EDF-13 and Sleep-EDF-18. Sleep-EDF-13 includes 61 full-night polysomnographic recordings containing EEG, EOG, chin EMG, and event markers, whereas Sleep-EDF-18 comprises 197 full-night recordings [27]. In this work, only the Fpz–Cz EEG channel was utilized. Following the latest AASM sleep staging criteria [28], the original N3 and N4 stages were combined into a single N3 stage [26,29]. The distribution of sleep stages across the two subsets is summarized in Table 3 [26,30].

Two key hyperparameters are involved in the MMD function: the width of the Gaussian kernel and the number of kernel functions [31]. A grid search strategy is adopted to identify hyperparameter settings suitable for the task. Specifically, 20% of the dataset is reserved as the test set, while the remaining 80% is split into training and validation sets at a 4:1 ratio. Model performance under different parameter combinations is then evaluated using five-fold cross-validation.

**Figure 12:**
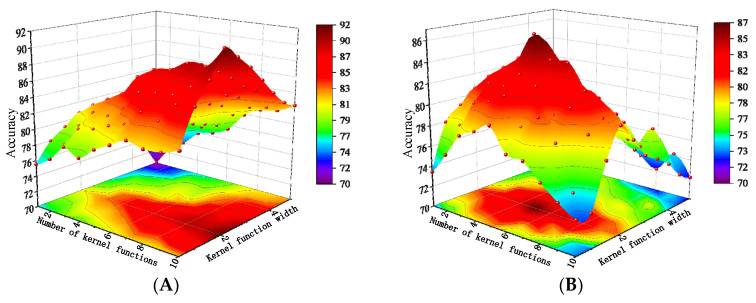
The results of various hyperparameter configurations and their corresponding accuracies. (**A**) Training set accuracy; (**B**) Validation set accuracy.

As shown in Figure 12, the model achieves the highest training accuracy when the Gaussian kernel width is set to 2 and the number of kernels is 10. For the validation set, the best accuracy is obtained with a kernel width of 2 and five kernels. To more intuitively illustrate the influence of each parameter on model accuracy, a precision trend chart based on the averaged accuracy is plotted.

**Figure 13:**
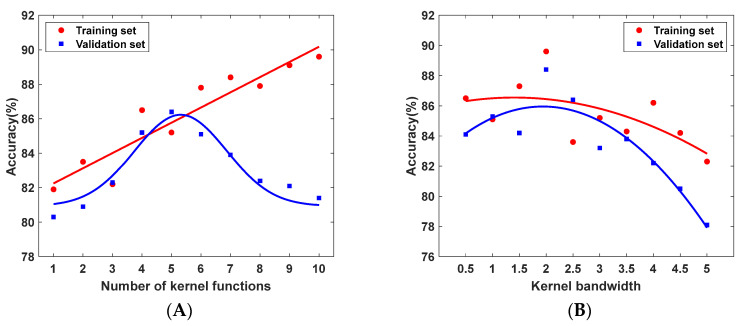
Trend chart based on the averaged accuracy. (**A**) Effect of the number of kernel functions on model accuracy; (**B**) Effect of the kernel width parameter on model accuracy.

As illustrated in Figure 13A, the training accuracy steadily increases with the number of kernel functions, whereas the test accuracy first improves and then declines. This pattern suggests that an excessive number of kernels increases model complexity, causing overfitting and a subsequent drop in accuracy. Figure 13B shows that when the Gaussian kernel width is set to 2, both the training and test sets achieve the highest accuracy, while widths smaller or larger than 2 result in a clear downward trend in performance.

#### 3.2.2. Comparative Analysis of Model Performance in Simulated Driving Environments

**Figure 14:**
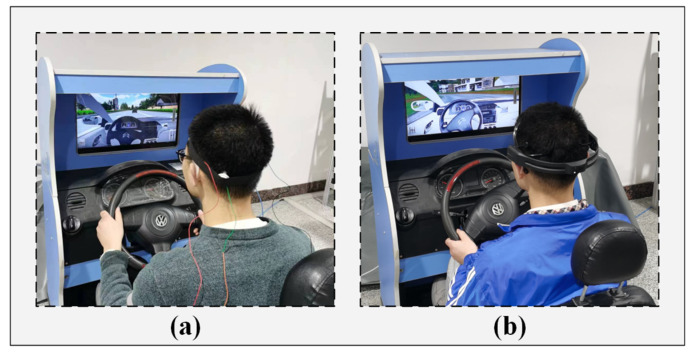
The environment diagram for the simulated driving scenario. (**a**) The electrode device proposed in this paper; (**b**) The Emotiv electrode device.

This performance data collection was conducted in a simulated driving environment, as depicted in Figure 14.

**Figure 15:**
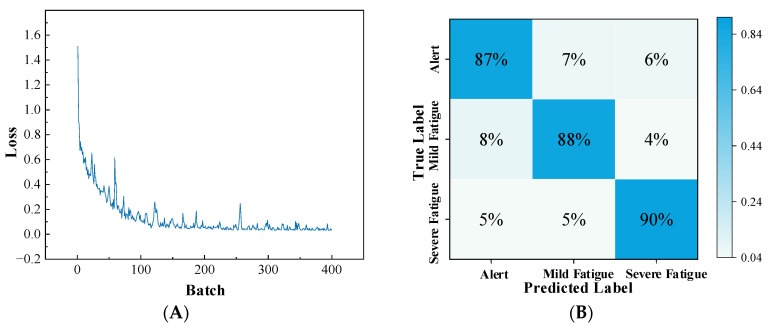
The transfer model loss curve and confusion matrix from training in the simulated driving environment. (**A**) Transfer model loss; (**B**) Transfer model confusion matrix.

As shown in Figure 15A, the loss decreases steadily and converges after around 160 epochs, suggesting that the model has effectively extracted features and achieved domain adaptation, thereby exhibiting strong classification capability. Meanwhile, the confusion matrix in Figure 15B further confirms the overall satisfactory performance of the model.

Additionally, five-fold cross-validation is conducted to assess model performance, and the corresponding results are summarized in Table 4. The comparative analysis of different metrics shows that the model performs consistently well across various dimensions, allowing reliable identification of driving fatigue states. Moreover, the small variance values highlight the model’s stability and its strong adaptability to heterogeneous datasets.

**Figure 16:**
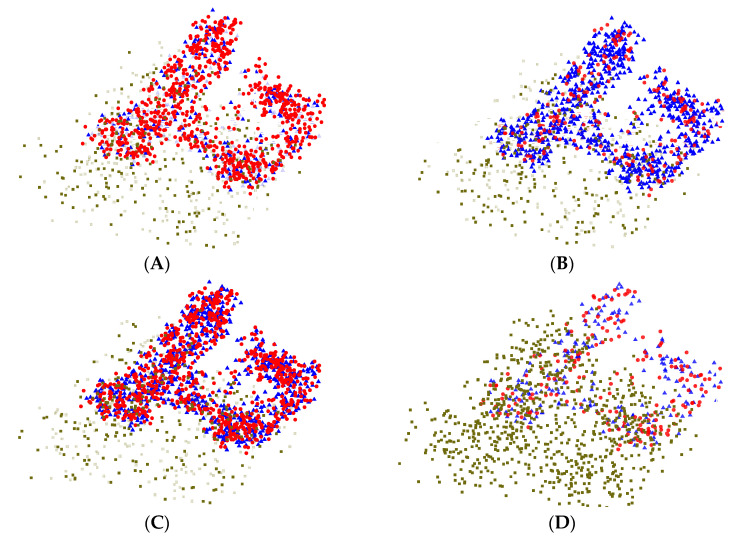
Visualization of the feature maps after processing. (**A**) Features extracted by the pre-trained model; (**B**) Features extracted by the MMD model; (**C**) Features extracted by both the pre-trained model and the MMD model; (**D**) Features extracted by the fine-tuned model. In the figure, circles represent features extracted by the pre-trained model, triangles represent features extracted by the MMD model, and squares represent features extracted by the fine-tuned model.

To better illustrate the generalization ability of the MMD model, the extracted features are visualized, as presented in Figure 16.

As illustrated in Figure 16, the features extracted by the pre-trained model show strong consistency with those obtained from the MMD model in the visual space. This suggests that the MMD model effectively leverages the knowledge acquired during pre-training to extract highly discriminative features.

This result is consistent with the theoretical foundation of the MMD approach. By minimizing the distributional discrepancy between the source and target domains, the model effectively mitigates the domain shift, thereby maintaining strong feature consistency and robustness across diverse data distributions.

In contrast, the Fine-tune model, which relies solely on parameter adjustment, produces features that differ considerably from those of the source domain, indicating limited ability to capture key representations compared with the MMD model. This comparison underscores the importance of applying domain adaptation techniques in transfer learning.

To further validate the advantages of the proposed model, Sleep EEG Net, the same experimental framework is applied to traditional neural networks, including CNN-1D, AlexNet, and ResNet-50.

**Figure 17:**
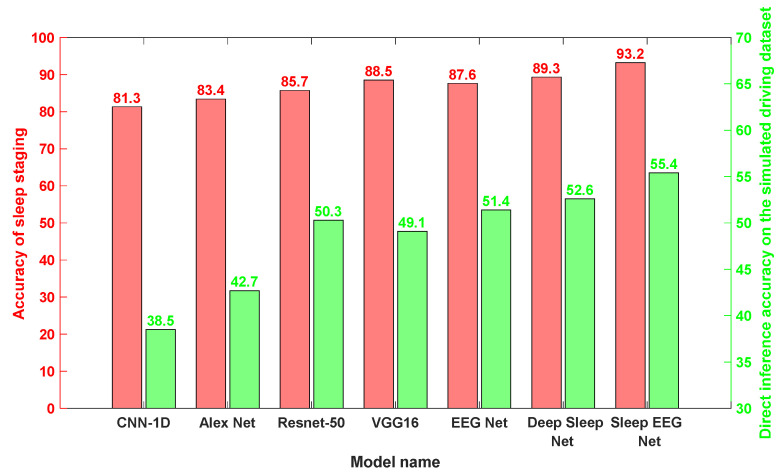
The accuracy achieved by each network under pre-training and direct transfer conditions.

As shown in Figure 17, all networks display reasonable effectiveness in sleep stage classification. Nevertheless, their performance drops noticeably when transferred directly to the task of drowsy driving detection. Even so, these models retain a moderate level of accuracy in the target domain, suggesting the presence of partially shared features between the two domains.

In this work, two transfer learning strategies—Fine-Tune and MMD—were applied. Their outcomes were benchmarked against domain-specific EEG models, namely EEGNet and Deep Sleep Net, as well as the proposed Sleep EEG Net. The detailed performance metrics of each transfer model within the simulated driving environment are summarized in Table 5.

The results in Table 5 reveal that transfer learning with EEGNet yields suboptimal performance, especially for the intermediate class of mild fatigue. By contrast, both Deep Sleep Net and the Sleep EEG Net employed in this study achieve similar and more reliable outcomes, indicating that long–short term neural network structures hold advantages in EEG signal processing.

Furthermore, the comparative analysis of chart data highlights marked performance gains in the transferred models, underscoring the effectiveness of transfer learning in bridging sleep staging tasks with drowsy driving detection.

#### 3.2.3. Comparative Analysis of Model Performance in Real Driving Environments

To further assess real-world applicability, this study tested the model’s generalization ability using EEG data collected from actual driving scenarios and compared its performance with a fine-tuned baseline.

**Figure 18:**
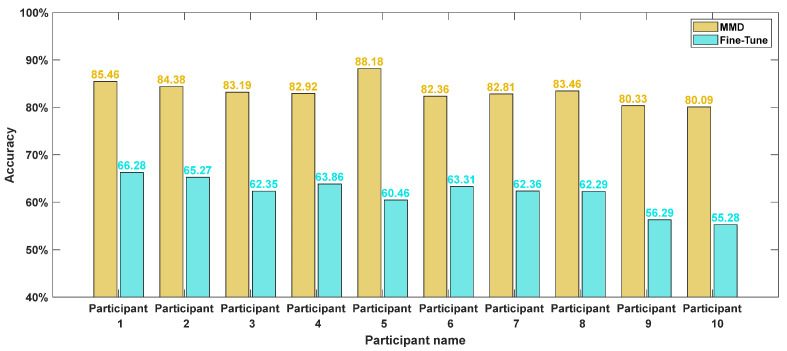
The accuracy rates of each models.

As illustrated in Figure 18, the MMD model demonstrates reliable recognition of driving fatigue in real driving conditions. Across ten participants, the model achieved an average accuracy of 83.3% with a standard deviation of 2.4, confirming its robustness and stability.

The EEG data collected from real driving environments was directly fed into the transfer models trained in the previous section for comparative evaluation. The training and test sets referenced correspond to the simulated driving datasets used during model transfer training.

**Figure 19:**
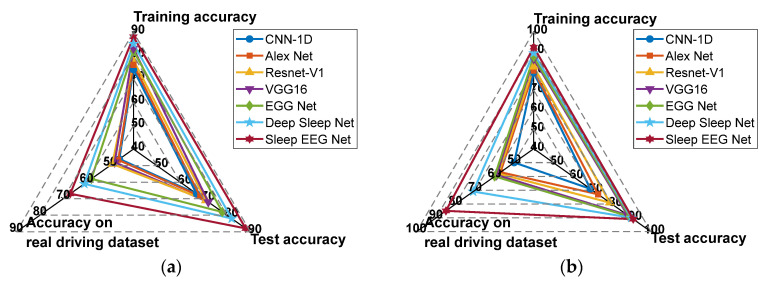
The accuracy rates of each model. (**a**) Recognition accuracy of each model under fine-tune transfer; (**b**) Recognition accuracy of each model under MMD transfer.

Based on the results presented in Figure 19, Sleep EEG Net consistently outperforms the other models across all evaluation metrics. Its superiority can be attributed to its sequence-to-sequence architecture tailored for sleep staging, as well as the integration of attention mechanisms, which enhance its ability to capture and recognize continuous temporal signals.

Furthermore, a comparison of Figure 19a,b shows that all transfer models incorporating MMD achieve notable improvements in recognition accuracy on the supplementary test set. These findings indicate that, relative to Fine-tune models, MMD-based approaches are more effective at leveraging features from both the source and target domains. Moreover, the proposed Sleep EEG Net demonstrates stronger generalization capability under these conditions.

The results in Table 6 show that when applied to real driving environments, the performance of all models declined to varying extents, with the most notable drop observed in the recognition of awake states. This reduction is likely due to higher levels of noise interference in real-world conditions, where drivers may perform additional physical actions—such as head movements to monitor traffic—compared to simulated environments where participants only focus on a screen, resulting in fewer electromyographic artifacts. Among the models, EEGNet exhibited the sharpest performance decline, suggesting that the features it extracts are not broadly generalizable. In contrast, Deep Sleep Net and Sleep EEG Net showed relatively stable performance, particularly in identifying mild fatigue, highlighting their stronger ability to capture contextual dependencies within continuous EEG time-series data.

To further assess category-specific performance, feature visualization was employed to compare models trained with MMD-based transfer learning.

**Figure 20:**
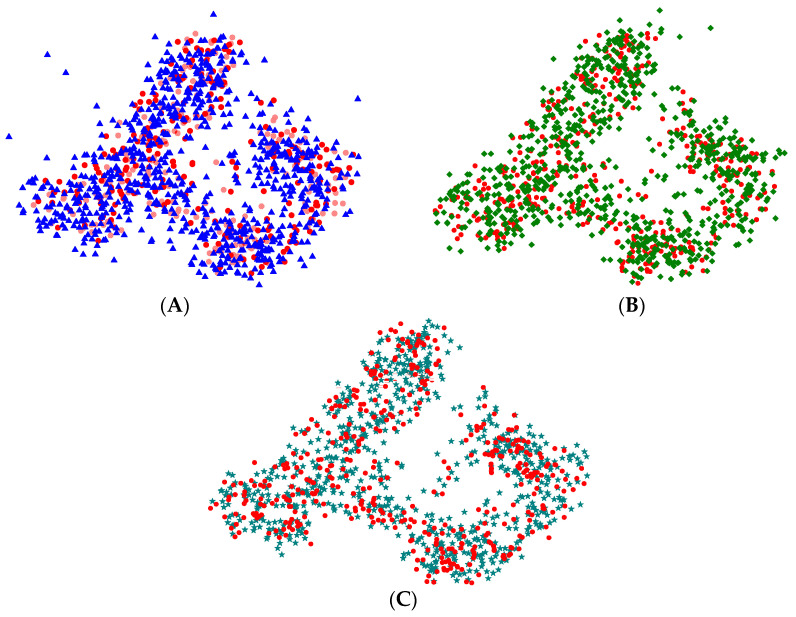
Visualization of the feature maps after processing. (**A**) Features extracted by the pre-trained model and EEGNet; (**B**) Features extracted by the pre-trained model and Deep Sleep Net; (**C**) Features extracted by the pre-trained model and Sleep EEG Net. In the figure, circles represent features extracted by the pre-trained model, triangles represent features extracted by EEGNet, diamonds represent features extracted by Deep Sleep Net, and pentagrams represent features ex-tracted by Sleep EEG Net.

As shown in Figure 20, the features extracted by all models achieve a general degree of alignment. Nevertheless, EEGNet and Deep Sleep Net display cases where certain intra-class features remain misaligned, leading to a relatively large number of outliers. Moreover, at the boundaries between clusters, EEGNet produces more dispersed inter-cluster features, whereas Deep Sleep Net performs somewhat better in this regard. These observations suggest that both models have limited ability to capture transitional features. In contrast, Sleep EEG Net exhibits more effective inter-cluster feature alignment, demonstrating stronger capability in handling transitional characteristics.

Furthermore, this observation is consistent with the architectural design of SleepEEGNet, which integrates multi-scale convolutional modules and attention mechanisms. These components enhance the network’s capacity to capture both local and global representations, thereby maintaining discriminative consistency within the feature space even when encountering cross-stage or boundary samples. Consequently, SleepEEGNet demonstrates superior generalization and robustness compared to conventional models.

## 4. Discussion

### 4.1. Semi-Dry Electrode with Automatic Electrolyte Replenishment

Traditional wet electrodes are widely used for EEG signal collection in static environments. They rely on conductive gel to lower the contact impedance between the skin and electrodes, thereby improving signal quality. However, this process is cumbersome, as the application of conductive liquid is time-consuming and labor-intensive, requiring manual operation, and the effective duration of each use is severely limited [32]. Once the gel dries, it must be reapplied, which may displace electrode positions and degrade signal quality [33]. In addition, to maximize the gel’s effectiveness, subjects are often required to undergo scalp exfoliation at electrode contact points. This procedure can cause skin discomfort, increase both labor and time costs, and demand considerable expertise from experimenters. After experiments, subjects must also clean residual gel from their hair, creating significant inconvenience. These limitations restrict the suitability of wet electrodes for wearable EEG acquisition. To overcome the drawbacks associated with conductive gel, researchers pioneered dry electrodes in the 1990s, eliminating the need for conductive liquids altogether.

In contrast, dry electrodes, first introduced in the 1990s, eliminate conductive gel altogether. They simplify preparation and improve wearability, requiring neither scalp exfoliation nor post-experiment cleaning. However, the absence of an electrolyte medium results in weaker skin adhesion, higher impedance, and greater vulnerability to power-line interference and motion artifacts.

More recently, semi-dry electrodes have emerged as a promising compromise, offering better stability, comfort, and convenience than either wet or dry types. Nonetheless, current semi-dry designs still depend on manual replenishment of conductive fluid. To address this limitation, this paper proposes a novel structural mechanism capable of automatically refilling fluid once it begins to dry. The mechanism remains sealed during inactivity to prevent leakage and activates only when replenishment is required. This innovation extends electrode lifespan, minimizes signal degradation from manual adjustments, and ensures more consistent EEG acquisition.

For the electrode–skin interface, traditional rigid metallic materials such as gold, silver, copper, or Ag/AgCl often cause discomfort and fail to fully conform to skin surfaces, compromising stability. In this study, a PVA sponge is introduced as the contact medium. Its high conductivity and deformability provide both reliable signal acquisition and improved user comfort by adapting closely to skin contours and reducing localized pressure.

A detailed comparison of electrode parameters is presented in Table 7.

### 4.2. Fatigue Detection Method

Traditional fatigue detection algorithms such as SVM exhibit certain limitations under small-sample conditions. As a discriminative model, SVM’s performance depends on sufficient and representative training samples. When the sample size is limited, SVM struggles to fully capture the underlying data distribution, often resulting in overfitting or degraded generalization performance, which ultimately reduces the classification accuracy of EEG signals.

To address these shortcomings in small-sample scenarios, this paper adopts a transfer learning approach based on MMD. By minimizing the distributional differences between source and target domains, the MMD method enables inter-domain alignment with limited data, thereby enhancing the stability and transferability of feature representations. Unlike traditional non-transfer algorithms, the MMD approach extracts robust distributional features without relying on large-scale labeled training data, thus maintaining high classification accuracy even when target domain samples are scarce or unlabeled.

The specific parameter comparisons of the algorithms are presented in Table 8 below.

### 4.3. Limitations and Future Prospects

The semi-dry electrodes designed in this study experience performance degradation or fatigue damage in the degreased hair components after repeated stretching, which may compromise their long-term stability. In addition, the magnets’ magnetic properties require precise control, potentially leading to slightly higher manufacturing costs compared to conventional electrodes. Future work will focus on identifying novel materials to replace degreased hair, with the goal of improving the durability and stability of the electrodes. Owing to the use of elastic bands in the electrode design, certain discomfort may be experienced by the subjects during use. In future work, the electrode structure will be further refined to appropriately reduce the pressure applied to the scalp while maintaining the quality and stability of signal acquisition.

## 5. Conclusions

This study proposes a semi-dry electrode with automatic conductive fluid replenishment for real-time and precise monitoring of driving fatigue. By integrating magnets with degreased hair, the electrode achieves autonomous fluid regulation, addressing the limitations of conventional electrode designs. Experimental validation demonstrates the electrode’s clear advantages, including improved fluid replenishment capability, prolonged operational lifespan, and enhanced wearing comfort. In addition, the use of flexible PVA sponge as the core skin-contact material effectively mitigates the physiological discomfort typically associated with traditional electrodes. On the algorithmic side, this research applies transfer learning techniques to fatigue state recognition, exhibiting robust generalization and effective cross-domain adaptability. Despite challenges such as limited data availability and stringent real-time requirements, the algorithm achieves rapid and accurate detection of driving fatigue. Overall, this work contributes to the development of stable and efficient fatigue monitoring technologies for the automotive sector, with the ultimate goal of enhancing driver safety and reducing potential traffic risks.

## Figures and Tables

**Table 1:** Parameters of the Feature Extractor.

Layers	Small Filter			Large Filter			Activate
	Window Size	Kernel Size	Stride	Window Size	Kernel Size	Stride	
Conv1d		50 × 1 × 64	6		400 × 1 × 64	50	ReLU
Maxpool	8		8	4		4	
Conv1d		8 × 1 × 128	1		6 × 1 × 128		ReLU
Conv1d		8 × 1 × 128	1		6 × 1 × 128		ReLU
Conv1d		8 × 1 × 128	1		6 × 1 × 128		ReLU
Maxpool	4		4	2		2	

**Table 2:** Parameters of Seq2seq.

Module	Layer Type	Parameters
Embedding	Dense lookup	embed_size = 10
Encoder	BiLSTM × 2	units = 128
Attention	Luong	attention_size = 64
Decoder	LSTM × 2	units = 128
Output Layer	Dense	5classes → 3classes
Loss	Sequence_loss + MMD + L2	kernel_num = 5, kernel_width = 2, β = 0.001
Optimizer	RMSProp	lr = 0.001

**Table 3:** Detailed information on the number of sleep stages in each version of the Sleep-EDF dataset.

Dataset	W	N1	N2	N3	REM	Total
Sleep-EDF-13	8285	2804	17,799	5703	7717	42,308
Sleep-EDF-18	65,951	21,522	96,132	13,039	25,835	222,479

**Table 4:** Performance Metrics of Transfer-Learned Models.

	Accuracy	Precision	Specificity	Recall	F1
Fold 1	0.9148	0.8778	0.9256	0.9042	0.8685
Fold 2	0.9039	0.8524	0.9547	0.8793	0.8568
Fold 3	0.9027	0.8623	0.8552	0.8921	0.8501
Fold 4	0.8793	0.8401	0.8467	0.9072	0.8331
Fold 5	0.8792	0.8547	0.8994	0.9018	0.8278
Mean	0.8877	0.8561	0.8943	0.8983	0.8488
Variance	0.0003	0.0004	0.0012	0.0002	0.0004

**Table 5:** Performance Metrics of Various Transfer Models Across Different Categories in Simulated Driving Environments.

		Prediction Label			Performance Metric (%)	
		Awake	Mild Fatigue	Severe Fatigue	Precision	Recall
EEGNet-FT	Awake	1768	892	421	59.9	57.4
	Mild Fatigue	593	962	658	37.4	43.5
	Severe Fatigue	591	717	2002	65.0	60.5
EEGNet-MMD	Awake	2014	641	430	75.9	65.3
	Mild Fatigue	354	1361	510	49.6	61.2
	Severe Fatigue	285	741	2269	70.7	68.9
Deep Sleep Net-FT	Awake	2017	621	662	71.0	61.1
	Mild Fatigue	431	1372	421	53.6	61.7
	Severe Fatigue	394	568	2341	68.4	70.9
Deep Sleep Net-MMD	Awake	2335	519	417	87.1	71.4
	Mild Fatigue	205	1901	124	70.8	85.2
	Severe Fatigue	141	266	2890	84.2	87.7
Sleep EEG Net-FT	Awake	2271	434	380	80.8	73.6
	Mild Fatigue	198	1852	174	74.3	83.3
	Severe Fatigue	341	205	2757	83.3	83.5
Sleep EEG Net-MMD	Awake	2685	211	187	88.8	87.1
	Mild Fatigue	175	1952	91	83.9	88.0
	Severe Fatigue	162	163	2970	91.4	90.1

**Table 6:** Performance Metrics of Various Transfer Models Across Different Categories in Real-World Driving Environments.

		Prediction Label			Performance Metric (%)	
		Awake	Mild Fatigue	Severe Fatigue	Precision	Recall
EEGNet-FT	Awake	851	654	471	56.3	43.1
	Mild Fatigue	366	422	285	31.2	39.3
	Severe Fatigue	294	276	706	48.3	55.3
EEGNet-MMD	Awake	908	647	425	61.9	45.9
	Mild Fatigue	401	491	175	33.2	46.0
	Severe Fatigue	158	340	787	56.7	61.2
Deep Sleep Net-FT	Awake	1008	658	313	64.9	50.9
	Mild Fatigue	402	519	144	35.1	48.7
	Severe Fatigue	142	303	825	64.4	65.0
Deep Sleep Net-MMD	Awake	1241	491	251	67.2	62.6
	Mild Fatigue	413	547	112	44.6	51.0
	Severe Fatigue	192	189	897	71.2	70.2
Sleep EEG Net-FT	Awake	1448	401	132	74.4	73.1
	Mild Fatigue	324	602	143	50.2	56.3
	Severe Fatigue	173	197	902	76.6	70.9
Sleep EEG Net-MMD	Awake	1725	142	103	87.3	87.6
	Mild Fatigue	198	767	99	77.1	72.1
	Severe Fatigue	53	86	1141	85.0	89.1

**Table 7:** Performance Comparison of the Novel Semi-Dry Electrode Developed in This Study with Other Conventional Electrodes.

Type of Electrodes	Impedance Value (at 10 Hz)	Signal-to-Noise Ratio (dB)	Time Available for Continuous Use	Comfort Rating	Whether the Conductive Fluid Can be Automatically Replenished
Emotiv wet electrode [34]	7.02 kΩ	12.86	2 h	5.45	No
Ag/AgCl wet electrode [35]	6.88 kΩ	13.37	2 h	5.89	No
Dry contact electrode [36]	15.68 kΩ				
Ceramic semi-dry electrode [12]	9.89 kΩ		10 h		No
Flexible multi-layer semi-dry electrode [15]	9.46 kΩ		5 h		No
Portable semi-dry electrode [37]	11.23 kΩ	11.13	7 h	5.14	No
Novel electrode (This study)	8.35 kΩ	12.28	16 h	5.42	Yes

**Table 8:** Comparison of the Algorithm Used in This Paper with Other Non-Transfer Learning Algorithms.

Dataset	Task Type	SVM (Mean ± Std)	MMD (Mean ± Std)
Self-built Dataset Cross-day [15]	Two classifications	0.7002 ± 0.159	0.7997 ± 0.153
SEED Cross-day [15]	Four classifications	0.5884 ± 0.1142	0.6817 ± 0.1350
Self-built Dataset Cross-subject [15]	Two classifications	0.6726 ± 0.147	0.7837 ± 0.151
SEED Cross-subject [15]	Four classifications	0.5818 ± 0.1385	0.6655 ± 0.0483
This Study’s Dataset	Two classifications	0.6925 ± 0.128	0.8032 ± 0.144

## Data Availability

No new data were created or analyzed in this study.

## Abbreviations

The following abbreviations are used in this manuscript:

EEG	electroencephalography
MF-DFA	multifractal detrended fluctuation analysis
BPNN	backpropagation neural network
RF	random forest
CNN	convolutional neural network
SVM	support vector machine
GADF-CNN	gauss-annex-difference field-convolutional neural network
KSS	Karolinska sleepiness scale
DDC	deep domain confusion
MMD	maximum mean discrepancy
MATL-DC	multi-domain aggregation transfer learning with domain-class prototype
TDDPL	transfer discriminative dictionary pair learning
RKHS	reproducing kernel hilbert space
